# Media audit reveals inappropriate promotion of products under the scope of the International Code of Marketing of Breast-milk Substitutes in South-East Asia

**DOI:** 10.1017/S1368980016003591

**Published:** 2017-03-15

**Authors:** Kristine Hansen Vinje, Linh Thi Hong Phan, Tuan Thanh Nguyen, Sigrun Henjum, Lovise Omoijuanfo Ribe, Roger Mathisen

**Affiliations:** 1 Department of Nursing and Health Promotion, Oslo and Akershus University College of Applied Sciences, Postboks 4, St. Olavs Plass, 0130 Oslo, Norway; 2 Alive & Thrive Southeast Asia, Hanoi, Vietnam; 3 FIAN Norway, Oslo, Norway

**Keywords:** International Code of Marketing of Breast-milk Substitutes, Market size and growth, Media audit, South-East Asia

## Abstract

**Objective:**

To review regulations and to perform a media audit of promotion of products under the scope of the International Code of Marketing of Breast-milk Substitutes (‘the Code’) in South-East Asia.

**Design:**

We reviewed national regulations relating to the Code and 800 clips of editorial content, 387 advertisements and 217 Facebook posts from January 2015 to January 2016. We explored the ecological association between regulations and market size, and between the number of advertisements and market size and growth of milk formula.

**Setting:**

Cambodia, Indonesia, Myanmar, Thailand and Vietnam.

**Results:**

Regulations on the child’s age for inappropriate marketing of products are all below the Code’s updated recommendation of 36 months (i.e. 12 months in Thailand and Indonesia; 24 months in the other three countries) and are voluntary in Thailand. Although the advertisements complied with the national regulations on the age limit, they had content (e.g. stages of milk formula; messages about the benefit; pictures of a child) that confused audiences. Market size and growth of milk formula were positively associated with the number of newborns and the number of advertisements, and were not affected by the current level of implementation of breast-milk substitute laws and regulations.

**Conclusions:**

The present media audit reveals inappropriate promotion and insufficient national regulation of products under the scope of the Code in South-East Asia. Strengthened implementation of regulations aligned with the Code’s updated recommendation should be part of comprehensive strategies to minimize the harmful effects of advertisements of breast-milk substitutes on maternal and child nutrition and health.

Optimal breast-feeding practices demonstrate lifelong benefits on the nutrition and health status of mothers and children, regardless of their socio-economic status^(^ ^1^ ^)^. Breast-feeding is recommended exclusively in the first 6 months and continued at 2 years of age^(^ ^2^ ^,^ ^3^ ^)^. By adhering to international breast-feeding recommendations, more than 800 000 deaths of children under 5 years of age and 20 000 deaths of women from breast cancer could be prevented annually^(^ ^1^ ^)^. Adopting optimal breast-feeding practices would prevent 12 400 child and maternal deaths each year in seven countries in South-East Asia (Cambodia, Indonesia, Laos, Myanmar, Thailand, Timor-Leste and Vietnam)^(^ ^4^ ^)^. Optimal breast-feeding practices also contribute to economic development through decreased expenditure on health and baby foods and a strengthened future workforce^(^ ^4^ ^–^ ^6^ ^)^. The WHO’s Global Nutrition Targets seek to increase the rate of exclusive breast-feeding in the first 6 months from 38 % to at least 50 % by 2025^(^ ^7^ ^)^.

Marketing of breast-milk substitutes (BMS) has a negative impact on optimal breast-feeding behaviours^(^ ^3^ ^,^ ^8^ ^)^ by altering knowledge, intention, beliefs, self-efficacy and social norms of mothers and other caregivers^(^ ^8^ ^,^ ^9^ ^)^. The International Code of Marketing of Breast-milk Substitutes (referred to hereafter as ‘the Code’) has guided the promotion and protection of breast-feeding over the last 35 years by setting provisions as a minimum standard to be adopted by nations^(^ ^10^ ^)^. The Code calls on individual governments to legislate and enforce the Code^(^ ^10^ ^)^. The Code is most effective when incorporated into legally enforceable measures and subsequently regulated, monitored and enforced at the national level^(^ ^3^ ^,^ ^11^ ^)^. The Code has been integrated (fully or partially) within legal documents in 135 and non-legal documents in forty-nine countries^(^ ^12^ ^)^. Four out of eleven countries in South-East Asia have integrated all provisions of the Code into national laws and regulations^(^ ^12^ ^)^. However, despite efforts to promote and protect breast-feeding, breast-feeding practices remain suboptimal in South-East Asia: early initiation of breast-feeding of less than 50 % and exclusive breast-feeding of 30 %^(^ ^13^ ^)^. There are large variations in breast-feeding practices in the region. For example, the exclusive breast-feeding prevalence was 74 % in Cambodia (in 2010), 24 % in Vietnam (in 2014) and 12 % in Thailand (in 2012)^(^ ^13^ ^)^.

Global sales of BMS were $US 44 billion in 2014 and have grown by nearly 10 % each year^(^ ^3^ ^)^. Rapid economic growth and a large child population make South-East Asia an important market for baby foods, including BMS^(^ ^14^ ^)^. To expand market size, BMS companies aggressively market their products in this region^(^ ^3^ ^,^ ^15^ ^)^ and growing-up milk (GUM; marketed for children over 12 months old) is the key product line, accounting for 50 % of market growth between 2013 and 2018^(^ ^14^ ^)^. Marketing of GUM contravenes the Code because it usually confuses mothers and family members about child age (i.e. a 12-month-old child in the advertisements could be perceived as a younger infant) and add-in nutrients or non-nutrients (i.e. closer to breast milk, but sounds like better than breast milk). To the detriment of public health, a global population-level infant and young child feeding transition towards diets higher in BMS is well underway and expected to continue, with the most rapid volume growth in the middle-income countries of East and South-East Asia, particularly in China, Indonesia, Malaysia, Thailand and Vietnam^(^ ^16^ ^)^.

The UN Convention on the Rights of the Child includes the right to adequate nutritious foods for children in order to combat disease and malnutrition and the right of parents to have access to information about the advantages of breast-feeding (Article 24)^(^ ^17^ ^)^. General Recommendation No. 34 of the Convention on the Elimination of all Forms of Discrimination against Women addressing the rights of rural women calls on all states to ensure broad dissemination of optimal health-care information through media outlets, including information on breast-feeding and the impact on child and maternal health^(^ ^18^ ^)^. States should also ensure effective regulation of the marketing of BMS and implementation and monitoring of the Code^(^ ^18^ ^)^. International, governmental and non-governmental organizations are providing assistance to countries in South-East Asia to strengthen the regional and national policies and environments to promote and protect breast-feeding in the region. However, to date, there is limited data related to mapping of national policies and status of violation of the Code in this region.

We conducted the present study to review regulations and to perform a media audit of marketing of products under the scope of the Code in Cambodia, Indonesia, Myanmar, Thailand and Vietnam. We then examined their association with market size and growth of milk formula in Indonesia, Thailand and Vietnam.

## Methods

### Definitions of terms

Marketing is defined as product promotion, distribution, selling, advertising, product public relations and information service^(^ ^10^ ^)^. BMS, also referred to as ‘milk formula’ by Euromonitor International, is defined as any food or beverage presented as a full or partial replacement for breast milk, whether or not suitable for that purpose^(^ ^10^ ^)^. The milk formula category includes standard milk formula for infants from 0 to 5 months; follow-on or follow-up milk formula for infants from 6 to 11 months; and toddler milk or GUM for young children aged from 12 months^(^ ^19^ ^)^. We also use ‘infant formula’ to refer to both standard milk and follow-on milk formula. Notably, these are not standardized categories; and BMS manufacturers and distributors use different age ranges and terms^(^ ^20^ ^)^. Feeding bottles and teats that fall under the scope of the Code, defined as any container with a teat meant to feed infants or young children^(^ ^10^ ^)^, were also included. Although milk for pregnant and lactating women has not been covered by the Code, we included it in the present study because of its similarities to the products under the milk formula category^(^ ^19^ ^)^. As international recommendations are that children are to be breast-fed until the age of 2 years or beyond, any milk formula could be considered as replacing breast milk until 36 months and therefore covered by the scope of the Code^(^ ^19^ ^)^.

### Data collection

#### National measures for implementation of the Code

We collected legal documents published online to identify an initial list of measures. We consulted with relevant experts to complete the list and then collected the full text of all of the policies. Non-English materials were translated into English before data analysis. For each policy, we qualitatively extracted information about the type of legal document, the products, age range and type of promotion under the scope of the regulation.

#### Mass media scanning of advertisements for breast-feeding and breast-milk substitutes

Between January 2015 and January 2016, independent media agencies were contracted to perform systematic media monitoring. The media scans lasted for 3 months in Vietnam and 6 months in Cambodia, Indonesia, Myanmar and Thailand (see online supplementary material, Supplemental Table 1). Keywords for the scan included breast-feeding, infant formula, follow-up or follow-on formula, toddler milk, growing-up milk, feeding bottles and teats, and milk for pregnant and lactating women, as well as brand names of popular products. Non-English materials were translated into English before data analysis. We targeted advertorials (defined as media materials combining information with product or brand promotion), editorial content (defined as all content produced by journalists; not performed in Myanmar) and Facebook posts (in Indonesia and Thailand). Companies and brand names identified through monitoring traditional media in Cambodia, Myanmar and Vietnam were chosen and the researchers collected the thirty latest posts from their Facebook pages. Company websites were not included in the data collection. We used open-accessed, de-identified data that were already collected and thus were exempt from institutional review board approval.

**Table 1:** National measures for implementation of provisions from the International Code of Marketing of Breast-milk Substitutes

Country	Measure	Legal status	Products included	Age range	Advertisements for BMS prohibited in general media	Marketing personnel prohibited to perform educational activities or to sponsor events	Distribution of gifts or samples prohibited
Cambodia	Sub-Decree on Marketing of Products for Infants and Young Child Feeding (in 2005)^(^ ^37^ ^)^ Related legislation: Joint Prakas 061 Implementation of Sub-Decree on Marketing of Products for Infant and Young Child Feeding (in 2007)*	Legally enforceable measure	Infant formula, Follow-on formula	0–23 months	✓	✓	✓
Indonesia	Regulation No. 33 on Exclusive Breastfeeding (in 2012)^(^ ^38^ ^)^ Related legislation: Act No. 36 on Health (in 2009)	Legally enforceable measure	Infant formula	0–11 months	✓	✓	–
	Regulation No. 39 on Infant Formula Milk and Other Baby Products (in 2013)^(^ ^39^ ^)^	Legally enforceable measure	Infant formula, Follow-on formula†	0–11 months	✓	✓‡	✓
Myanmar	Order of Marketing of Formulated Food for Infant and Young Children (in 2014)^(^ ^40^ ^)^ Related legislation: National Food Law (in 1997, amended in 2014)	Legally enforceable measure	Infant formula, Follow-on formula	0–23 months	✓	✓	✓
Thailand	Code of Marketing of Foods for Infants and Young Children and Related Products (in 2008)	Voluntary	Infant formula, Follow-on formula	0–11 months	✓	–	✓
Vietnam	Decree on Trading in and Using of Nutritional Products for Infants, Feeding Bottles and Dummies (in 2014)^(^ ^41^ ^)^ Related legislation: Law on Children Protection, Care and Education	Legally enforceable measure	Infant formula, Follow-on formula	0–23 months	✓	✓	✓
	Law on Advertisement (in 2012)^(^ ^42^ ^)^	Legally enforceable measure	Infant formula, Follow-on formula	0–23 months	✓	✓	✓

* Responsibilities of the implementation of the Sub-Decree are clarified.

† Referred to as ‘baby products including milks’.

‡ Prohibitions to accept sponsoring.

#### Market size and growth of milk formula

We purchased data about market size (i.e. the annual sales) and annual market growth of milk formula in Indonesia, Thailand and Vietnam from Euromonitor International (http://www.euromonitor.com). Similar data for Cambodia and Myanmar were not available.

### Analysis

#### National measures for implementation of the Code

The following articles of the Code were selected to analyse and assess compliance by countries and companies: Article 4 on information and education; Article 5 on the general public and mothers; Article 8.2 on employees of manufacturers and distributors; and Article 11 on implementation^(^ ^10^ ^)^. National measures related to the Code were reviewed to assess regulations operating in the studied countries. The assessment focused on the legislative status (voluntary or legally enforceable) of related measures, the products and age ranges covered, and the inclusion of BMS marketing limitations.

#### Media analysis

Text, pictures and audio-visuals were examined to identify key messages and issues, stakeholders involved and products that were promoted. Advertisement clips were categorized based on the industry categories of products previously listed. When an age range for a product was given, the lowest age was used. For example, a product said to be suitable for children aged 1–3 years was placed in category GUM from 12 months. For advertisements that did not include an age range of the product, we got the information from companies’ websites.

Total annual estimate of advertisements equals the identified number of advertisements multiplied by 4 in Vietnam, and by 2 for the other four countries. The estimation was based on the assumption that the advertisements were distributed equally by month for each country. The estimation is needed to enhance the comparability of findings from different countries (i.e. the duration of data collection was different) and to be used in the ecological analysis. We did not perform a similar analysis with materials relating to breast-feeding promotion because it would vary substantially with time (e.g. higher in August due to events relating to World Breastfeeding Week).

#### Compliance assessment

Obligations of governments and BMS manufacturers and distributors were addressed, with a specific focus on identifying violations and circumventions. Circumvention was defined as any practice not directly in violation of the Code, but in some way evading the restrictions. A type of circumvention considered in the present study was cross-promotion, defined as promotion of a product outside national regulations or the Code by making reference to a covered product via similarities in product design, labelling or colouring scheme^(^ ^20^ ^)^.

#### Ecological association between market size of milk formula and policies

For Indonesia, Thailand and Vietnam, we displayed the trend of market size of overall milk formula, standard milk formula, follow-on formula and GUM from 2000 to 2014 and with projection to 2016. Then, we plotted national policies against those trends.

#### Ecological association between market size and growth of milk formula and the number of advertisements

Then, we plotted the total annual estimate of advertisements (i.e. on television and in print) against market size in 2014 and market growth between 2013 and 2014 for milk formula, which took into account the country’s number of births in 2015^(^ ^21^ ^)^. We also fit a simple linear regression line with a coefficient of determination (*R* ^2^) to illustrate the direction and predictability of the number of advertisements on the market size and growth.

## Results

### National measures in the implementation of the Code

Five countries in the present study have adopted the Code (Table 1). Thailand is the only country with a voluntary agreement between the government and baby-food companies. In Cambodia, Myanmar and Vietnam, national measures regulate products for children up to 24 months. In Thailand and Indonesia, only BMS intended for feeding infants up to 12 months are covered. Indonesia has several regulations adopting different parts of the Code; the two regulations covering the scope of marketing in mass media were utilized for the present study. In Vietnam, the Advertisement Law (Article 7) became effective in 2012, and then the Decree was issued in 2014 to provide guidelines for the implementation and enforcement of this Law.

### Mass media coverage of and advertisements for breast-feeding and breast-milk substitutes

#### Advertisements

In total, 387 advertisements (in print, online or television) were collected (Table 2). Television was the main channel for advertisements in Cambodia and Vietnam; while print advertisements were more popular in the other three countries. We did not identify advertisements for formula milk for children under 12 months old. GUM accounted for 80 % of total advertisements, and was advertised as suitable for children above either 12 or 24 months (Table 2). However, the differentiation of the advertisements was based on national regulations (i.e. 12 months in Thailand and Indonesia and 24 months in the other three countries) for the same products. Milk for pregnant and lactating women was mentioned in about 10 % of the advertisements (Table 2), which claimed to benefit mothers, fetuses or newborns. GUM was usually advertised as meeting the requirements of growing children and helping them get taller and smarter.

**Table 2:** Advertisements for breast-milk substitutes, including bottles and teats and milk for pregnant and lactating women*, in five South-East Asian countries, January 2015 to January 2016

	Cambodia (*n* 117)		Indonesia (*n* 104)		Myanmar (*n* 44)		Thailand (*n* 84)		Vietnam (*n* 38)		Total annual estimate† (*n* 850)	
	*n*	%	*n*	%	*n*	%	*n*	%	*n*	%	*n*	%
By product/category‡												
Growing-up milk (≥12 months)	–	–	77	74·0	–	–	54	64·3	–	–	262	30·8
Growing-up milk (≥24 months)	110	94·0	–	–	32	72·7	–	–	34	89·5	420	49·4
Milk for pregnant and lactating women	7	6·0	8	7·7	12	27·3	12	14·3	3	7·9	90	10·6
Other brand or product promotion	–	–	19	18·3	–	–	18	21·4	1	2·6	78	9·2
By media channel												
Print	25	21·4	67	64·4	29	65·9	60	71·4	16	42·1	426	50·1
Television advertisements	92	78·6	37	35·6	15	34·1	24	28·6	22	57·9	424	49·9

* The duration for data collection was 3 months for Vietnam and 6 months for the other four countries.

† Total annual estimate equals identified number of advertisements multiplied by 4 in Vietnam, and by 2 for the other four countries.

‡ If breast-milk substitutes for several age ranges were promoted in the same advertisement, the lowest age was considered. If growing-up milk and milk for pregnant and lactating women were promoted in the same advertisement, they were accounted for in both categories; we did not find any violation for the product categories Standard milk formula and Follow-on formula (0–11 months).

#### Editorial content

BMS was mentioned in almost two-thirds of the total editorial content collected in Vietnam and about one-third in Cambodia, Indonesia and Thailand (Table 3). Articles were primarily published in lifestyle magazines targeting mothers and providing information about breast-feeding or bottle-feeding. The editorial content came from a variety of sources (e.g. independent journalists or non-specified editorial members) and was, in general, not aligned with recommendations given in Article 4 of the Code, which specifies what is to be included regarding breast-feeding information.

**Table 3:** Editorial content relating to breast-feeding and breast-milk substitutes* in five South-East Asian countries, January 2015 to January 2016

	Cambodia (*n* 22)		Indonesia (*n* 181)		Thailand (*n* 305)		Vietnam (*n* 292)		Total annual estimate† (*n* 2228)	
	*n*	%	*n*	%	*n*	%	*n*	%	*n*	%
By topic										
Breast-feeding	16	72·7	139	76·8	188	61·6	110	37·7	1158	52·0
Breast-milk substitutes	6	27·3	42	23·2	117	38·4	182	62·3	1070	48·0
By media channel										
Newspaper (print/online)	17	77·3	147	81·2	184	60·0	142	48·6	1298	58·3
Magazines (print/online)	5	22·7	34	18·8	122	40·0	150	51·4	932	41·8

* Editorial content not included in the media scanning in Myanmar. The duration for data collection was 3 months for Vietnam and Cambodia and 6 months in Indonesia and Thailand.

† Total annual estimate equals identified number of advertisements multiplied by 4 in Vietnam and Cambodia, and by 2 in Indonesia and Thailand.

#### Facebook posts

We collected 217 Facebook posts or conversations. BMS companies created and moderated online forums, in which mothers could share experiences or seek advice about infant and young child feeding. Common online conversations were about new mothers worrying about not being able to produce enough breast milk, questions around breast milk having sufficient energy and nutrients for newborns, pressure received from other family members to use BMS, and how to use formula and bottle-feeding. Supplementing with BMS was usually advised for mothers who perceived breast-milk insufficiency. Mothers generally agreed that formula-fed infants would gain more weight or grow better than exclusively breast-fed infants.

BMS companies also used their Facebook pages to promote their brands and products and often gave advice about infant and young child feeding. BMS for children under 24 months old was promoted either directly by using ambiguously the word ‘baby’ or indirectly by showing pictures of the products, newborns, infants or young children, or sales representatives or health workers offering free samples to new parents in hospitals. Parents were invited to like and share pictures online, and the companies gave advice about infant and young child feeding.

#### Violations and circumventions

Although monitored advertisements complied with child age in the national regulations, the advertisements of GUM violated Article 5.1 of the Code^(^ ^10^ ^)^ by promoting products that can partially or fully replace breast milk in the diet of children aged 0–36 months. Further violations of the Code were discovered in editorial content displaying women’s public events organized by milk companies. Such activities violated Articles 5.5 and 8.2^(^ ^10^ ^)^ of the Code, which prohibit marketing personnel from contacting mothers. In both advertisements and editorial content, there was widespread use of baby pictures and feeding bottles, thus cross-referencing products that fall under national prohibitions. Advertisements for milk for pregnant and lactating women might circumvent the Code by cross-promotion with the use of similar brands, labels and colour schemes to BMS.

### Ecological association between the trend of market size and policies

**Fig. 1:**
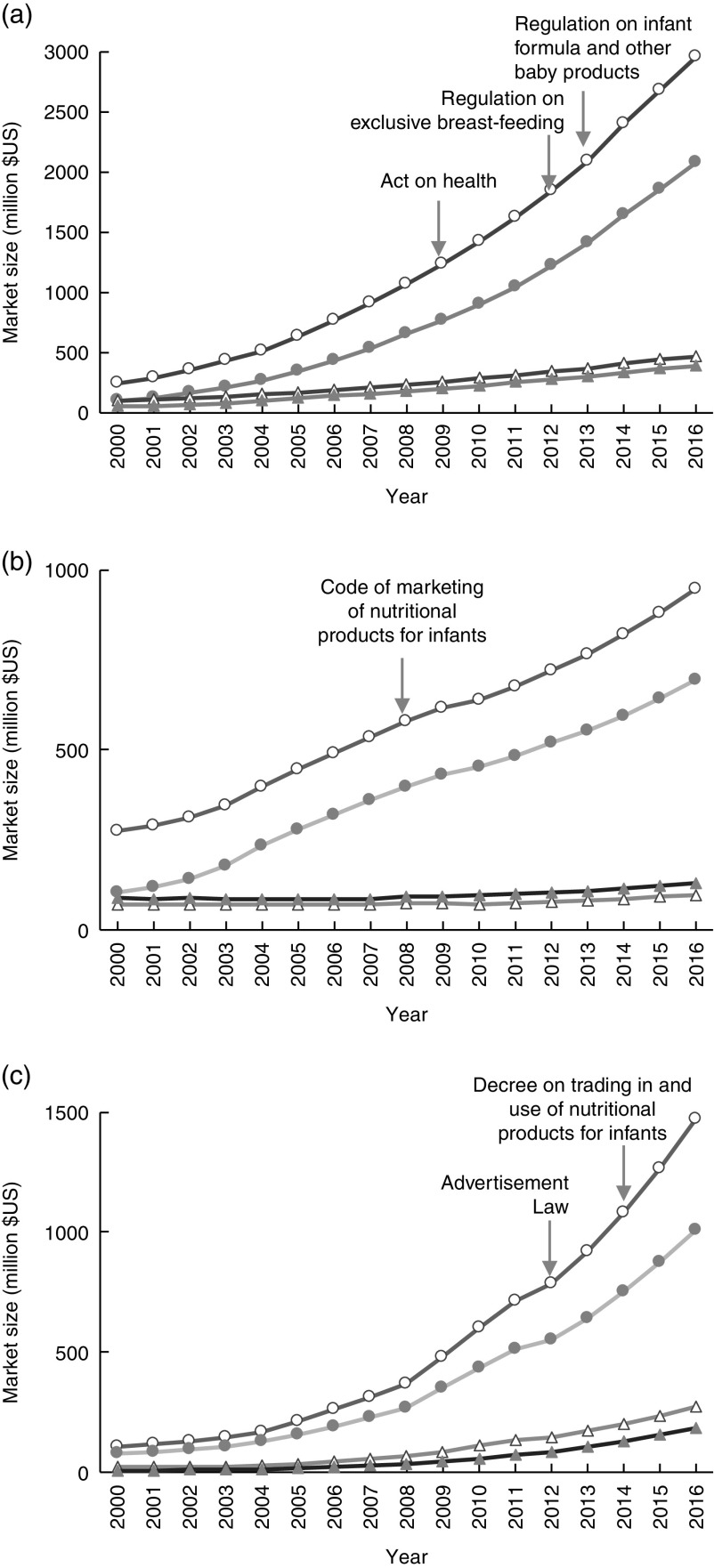
Market size for milk formula (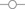, milk formula (overall); 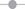, growing-up milk; 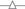, follow-on formula; 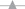, standard milk formula) in Indonesia (a), Thailand (b) and Vietnam (c). The market size was based on the Euromonitor report released in 2015^(^ ^43^ ^)^

The market size of milk formula is expanding in Indonesia, Thailand and Vietnam (Fig. 1). The value growth of market size between 2009 and 2014 was 125 % in Vietnam, 96 % in Indonesia and 33 % in Thailand. In the three countries, market size and growth were contributed largely by GUM (Fig. 1). There was some growth in market size for standard and follow-on milk formula in Vietnam and Indonesia, not in Thailand (Fig. 1). We did not see a clear association of laws and regulations with the market size trends (Fig. 1).

### Ecological association between market size and growth and advertisements

**Fig. 2:**
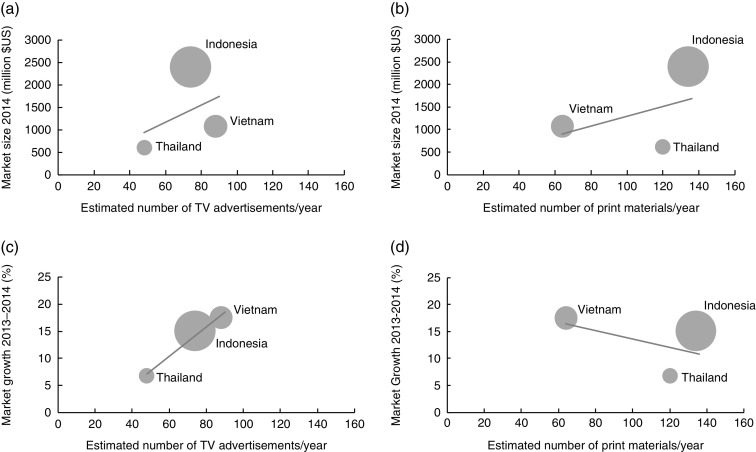
Association between market size of formula milk and annual number of television (TV) advertisements (a) and print materials (b), and association between market growth of formula milk and annual number of TV advertisements (c) and print materials (d). The size of the bubble indicates the 715 000, 5 037 000 and 1 582 000 newborns in 2015 in Thailand, Indonesia and Vietnam, respectively (based on UNICEF’s estimation^(^ ^21^ ^)^). The market size and growth were based on the Euromonitor report released in 2015^(^ ^43^ ^)^. (a) *y*=19·01*x*+32·337, *R* ^2^=0·1723; (b) *y*=11·08*x*+188·54, *R* ^2^=0·1949; (c) *y*=0·274*x*–6·0487, *R* ^2^=0·9814; (d) *y*=–0·0791*x*+21·516, *R* ^2^=0·2722

The number of television advertisements was highest in Vietnam and lowest in Thailand; while the number of print materials was highest in Indonesia and lowest in Vietnam (Fig. 2). There was a positive association between market size and the number of newborns and the number of television and print advertisements (Fig. 2). Annual market growth was positively associated with the number of television advertisements, but negatively associated with the number of print advertisements (Fig. 2).

## Discussion

Although the governments of Cambodia, Indonesia, Myanmar, Thailand and Vietnam have endorsed and implemented the Code, the marketing of BMS was numerous and non-compliant with the Code. We identified various tactics used by BMS companies to market their products. First, they specify GUM in the advertisements based on national regulations (i.e. the same GUM product was advertised for children above 12 months in Thailand and Indonesia, but for 24 months old in the other three countries). Second, they cross-promote their products by using similar branding, pictures and logos on a range of products from milk for pregnant and lactating women to GUM for children up to 36 months and beyond, which reduces the effectiveness of national restrictions on infant formula advertising^(^ ^22^ ^)^. Third, they claim unsubstantiated health benefits such as optimal development or contributing to stronger, taller and more intelligent children. In World Health Assembly Resolution 63.23 from 2010, governments are called upon to ensure that health claims are not permitted for foods for infants and young children^(^ ^23^ ^)^. A study in Cambodia found that almost half of mothers desired to feed their child BMS when they can afford it^(^ ^24^ ^)^ because they believed that BMS makes their children healthy and smart^(^ ^24^ ^)^. Fourth, they use social media and networking sites – an unregulated platform – to promote BMS, give advice and expand exposure to their products (i.e. by encouraging mothers to ‘like’ the posts and share their own experiences in the comment columns). They also sponsor health events that position BMS manufacturers and distributors as reliable sources for health information.

We audited Facebook pages because Facebook is the most frequently used social media platform in the studied countries^(^ ^25^ ^)^. We found that Facebook pages can be used as an unregulated platform for BMS manufacturers to promote all categories of BMS, share nutrition and breast-feeding information, and encourage parents to engage in digital conversations. Previous studies also indicated that companies might create and moderate web-based communities and use them as a vehicle to promote products and perform educational functions directed at the parents of infants and young children^(^ ^26^ ^,^ ^27^ ^)^. Mobile and web-based technologies track the behaviours of parents and offer an opportunity for companies to promote their products and interact directly with consumers^(^ ^26^ ^)^. Social media has been used by BMS companies as a platform to advertise their products and to create and strengthen bonds between companies and customers. Developing relationships were based on perceived trust, which cannot be done by traditional media^(^ ^28^ ^)^. Encouraging users to post comments, experiences or pictures provides brand sites with potential marketing content without requiring companies to break any Code rules (i.e. the proscription of the use of images idealizing artificial feeding)^(^ ^27^ ^)^. A wide and growing range of social media platforms are being used for BMS promotional activities^(^ ^27^ ^)^ and should therefore be included in Code monitoring activities.

It is not clear whether editorial content (i.e. created by journalists) is covered under Article 4 of the Code concerning breast-feeding information. A common interpretation of the Code would be that those provisions would pertain to information materials distributed at health-care facilities, which is not under the coverage of the present study. Still, Article 4.1 states that ‘governments should have the responsibility to ensure that objective and consistent information is provided on infant and young child feeding for use by families and those involved in the field of infant and young child nutrition’^(^ ^10^ ^)^.

Our study did not capture organized efforts by governments to disseminate information about breast-feeding via mass media. Mass media campaigns can be effective in changing behaviours, such as improving the desire to breast-feed^(^ ^29^ ^,^ ^30^ ^)^. It has been argued that breast-feeding should be ‘advertised’ through mass media to help mothers to access information, advice and support to promote optimal breast-feeding practices^(^ ^31^ ^)^. Mass media might also be used to raise awareness about the Code and national laws and regulations to promote and protect breast-feeding. Because the baby-food industry has greater financial resources and incentives, a recommended strategy for governments might be to stop the marketing of BMS rather than trying to outcompete the industry by increasing breast-feeding promotion. However, even if policies and programmes promoting breast-feeding have the potential to mitigate the negative impact of BMS marketing, they are not likely to completely reverse the influence that marketing may have had^(^ ^8^ ^)^. World Health Assembly Resolution 54.2 calls on governments to strengthen mechanisms to ensure Code compliance in all forms of media, emphasizing modern communication methods, including electronic means^(^ ^32^ ^)^. However, the present study shows a great need for better clarification of the Code’s provisions as they pertain to social media and for the World Health Assembly to provide recommendations and guidance for national implementation and enforcement of online prohibitions.

We did not find an association between milk formula sales and the effective date of laws and regulations. There are several potential explanations. First, BMS companies might sidestep the laws by various strategies such as cross-promotion, using Facebook, company websites, retail seller websites or promotion events, which are difficult to monitor to identify violations for enforcement. Second, companies are focusing on the growth of GUM, which is not under national regulations. Third, it takes time to detect a decrease in market size because: (i) women’s decisions or plans with regard to feeding their infant may be established before pregnancy, and early feeding of infant formula is associated with increased subsequent feeding of milk formula^(^ ^33^ ^)^; and (ii) it takes time to change the beliefs of caregivers and social norms related to feeding infant formula to newborns, infants and young children (i.e. breast milk is not sufficient at birth and for the growing child; it is best to combine breast milk with infant formula; infant formulas help child growth and brain development).

The complex relationship between the number of newborns, market size and growth, and the number of advertisements could be explained as follows. At the country level, although the number of newborns was associated with market size, market growth, however, was associated with the number of advertisements on television. This indicates that television advertisements would be a key strategy for expanding the market of a BMS company. In Vietnam, the number of print materials was lower than in Thailand. Potential explanations are that Vietnam has a quite intense television campaign already or that Thailand’s market was already dependent on milk formula and, thus, just needed periodic reminders to maintain market size. In Indonesia, the number of print materials was highest and the number of television advertisements was second highest. Because Indonesia had the largest number of newborns, there were notable numbers of advertisements on television and in print. However, because mothers were dependent on milk formula (more than Vietnamese mothers), there were fewer advertisements on television and more advertisements in print compared with Vietnam.

### Recommendations for policy action

Recommendations for protection and promotion of breast-feeding in legislation and policy making are summarized in Box 1. Because the Code only guides action, its adoption, implementation, monitoring and enforcement rely on government’s will^(^ ^3^ ^)^. As part of commitments made under the Convention on the Rights of the Child and the Convention on the Elimination of All Forms of Discrimination against Women^(^ ^17^ ^,^ ^18^ ^)^, mothers (and families) of infants and young children need to have access to consistent and unbiased information regarding infant nutrition and breast-feeding.

Box 1 Recommendations for protection and promotion of breast-feeding

Laws and regulations:

∙ Cover fully the Code and ensure strong enforcement against violations.
∙ Cover all products that substitute breast milk.
∙ Expand the scope of products to include those up to 36 months to align with updated regulations from UN General Assembly.
∙ Have strong monitoring and enforcement mechanisms, especially focusing on social media, cross-promotion and company-sponsored events.

Health professionals or government officials:

∙ Increase capacities to utilize mass media for breast-feeding protection and promotion.
∙ Engage journalists to report on breast-feeding topics.
∙ Ensure that media agencies are aware of the Code and regulations and clarify the roles and responsibilities of media agencies in charge of placing advertisements.

Mass media is a potential channel to influence the adoption of the Code and to improve breast-feeding practices^(^ ^29^ ^)^. By engaging with journalists, making them aware of the consequences and controversies that unethical marketing of BMS has and its effect on maternal and child health, journalists can act as ‘watchdogs’ and help report violations. In addition, enlisting the help of civil societies, non-governmental organizations, breast-feeding interest groups, mom bloggers, and web owners and administrators would play a role in identifying violations and creating social pressure to enhance compliance to the Code.

### Strengths and limitations

The use of professional media agencies for data collection has both strengths and limitations. Among the strengths of using a media monitoring service are that such agencies have developed efficient methods to capture and collect a large number of media clips across five South-East Asian countries. The data collection is not prone to recall bias, which may present an issue in retrospective studies or other Code monitoring activities where mothers are asked to recall breast-feeding variables or their exposure to BMS advertising^(^ ^34^ ^–^ ^36^ ^)^.

There were limitations of the present study as well. Most of the content needed to be translated into English by the media agencies before data analysis, which might not fully capture the advertisement contents and contexts. Different media agencies performed the media monitoring, which leads to a slight difference in data collection methods, platforms and sample selection. Furthermore, because the data collection was not at the same time in all countries, findings might not be fully comparable across countries.

### Research gaps and potential for further research

To confirm the study findings, more investigations of social media sites are needed. Likewise, pop-up advertisements on online sites and company websites should be addressed to map the extent of Code violations. To the knowledge of the authors, cross-promotion has been assessed only between the various categories of BMS, and not for milk for pregnant and lactating women. The influence of marketing of such products on the recommended breast-feeding behaviour should hence be further investigated. The average contract value for the media audit was about $US 2000 per month, which ranged from about $US 1000 in Vietnam to $US 3800 in Cambodia. The variation was depending on available media and market research platforms and services. Our study could provide a template for collecting, managing and extracting information from media, and thus reduce the cost of future study.

## Conclusions

The current media audit reveals inappropriate promotion and insufficient national regulation of products under the scope of the Code in South-East Asia. Strengthened implementation of regulations aligned with the Code’s updated recommendation should be part of comprehensive strategies to minimize the harmful effects of advertisements of BMS on maternal and child nutrition and health.

## Supplementary material

For supplementary material accompanying this paper visit https://doi.org/10.1017/S1368980016003591. click here to view supplementary material
