# Supplementary material for: Media audit reveals inappropriate promotion of products under the scope of the International Code of Marketing of Breast-milk Substitutes in South-East Asia
Source: Public Health Nutr. 2017 Mar 15;20(8):1333–42. doi: 10.1017/S1368980016003591 (PMC10261673; doi:10.1017/S1368980016003591)
Supplement: Supplementary file 1 [file S1368980016003591sup001.docx]

**Supplemental Table 1.** Timeline and content of policy reviews and media scan

| **Countries** | **National**  **Policies and Regulations*^1^*** | **Timeline of scanning** | | **The type of media scanned*^2^*** | | | | | |
| --- | --- | --- | --- | --- | --- | --- | --- | --- | --- |
|  |  | Data collection months | Total months | Television advertisements on BMS | Print materials on BMS | Editorial content on BMS | Editorial content on breastfeeding | Facebook online conversations*^3^* | Online conversations |
| Cambodia | V | Aug 2015 to Jan 2016 | 6 mo | ✓ | ✓ |  |  | ✓ |  |
|  |  | Aug and Oct 2015 and Jan 2016 | 3 mo |  |  | ✓ | ✓ |  |  |
| Indonesia | V | Jan to June 2015 | 6 mo | ✓ | ✓ | ✓ | ✓ | ✓ | ✓ |
| Myanmar | V | Mar to Aug 2015 | 6 mo | ✓ | ✓ |  |  | ✓ |  |
| Thailand | V | Jan to June 2015 | 6 mo | ✓ | ✓ | ✓ | ✓ | ✓ | ✓ |
| Vietnam | V | Aug to Oct 2015 | 3 mo | ✓ | ✓ | ✓ | ✓ | ✓ |  |

BMS – breastmilk substitutes

*^1^* For each policy, we qualitatively extracted information about type of legal document, products, age range, and type of promotion under the scope of the regulation.

*^2^* Data collected by media agencies; non-English materials were translated into English before data analysis. Researchers reviewed the media using structured evaluation form.

*^3^* Data collected by media agencies for Indonesia and Thailand; and by authors through Facebook of major BMS companies.
